# 0588. Effects of norepinephrine on tissue perfusion in a sheep model of intraabdominal hypertension

**DOI:** 10.1186/2197-425X-2-S1-P33

**Published:** 2014-09-26

**Authors:** G Ferrara, VS Kanoore Edul, JF Caminos Eguillor, E Martins, C Canullán, HS Canales, C Ince, A Dubin

**Affiliations:** Facultad de Ciencias Médicas, Universidad Nacional de La Plata, Cátedra de Farmacología Aplicada, La Plata, Argentina; Academic Medical Center, Department of Translational Physiology, Amsterdam, Netherlands

## Introduction

Intraabdominal hypertension (IAH) produces detrimental effects on tissue perfusion. A putative underlying mechanism is the decrease in abdominal perfusion pressure (APP = mean arterial pressure-intraabdominal pressure). Nevertheless, the benefits of increasing blood pressure on tissue perfusion are controversial.

## Objectives

To describe the effects of IAH on regional and microcirculatory intestinal blood flow, renal blood flow, and urine output, as well as their responses to increases in blood pressure induced by norepinephrine.

## Methods

In 24 anesthetized, mechanically ventilated, and fluid-resuscitated sheep, we measured systemic hemodynamics, left renal and superior mesenteric artery blood flows, villi microcirculation, ileal intramucosal-arterial PCO_2_ (ΔPCO_2_), and urine output. IAH (20 mm Hg) was generated by intraperitoneal instillation of warmed saline. After 1 h of IAH, sheep were randomized to control (n = 8) or norepinephrine (n = 8) groups for 1 h. In this last group, mean arterial pressure was increased about 20 mm Hg by means of norepinephrine. A sham group (n = 8) was also studied.

**Table 1:** Table 1

Period	Group	APP (mm Hg)	Cardiac output (mL.min^-1^.kg^-1^)	Mesenteric flow (mL.min^-1^.kg^-1^)	ΔPCO_2_ (mm Hg)	Villi perfused density (mm/mm^2^)	Renal flow (mL.min^-1^.kg^-1^)	Urine output (mL.min.kg^-1^)
Basal	Control	83±12	122±26	392±154	7±6	22±3	1906±517	1.2±0.3
	Norepinephrine	82±7	96±17	445±318	11±7	26±3	1905±729	1.1±0.5
	Sham	76±13	113±18	405±115	4±4	23±4	1890±639	1.4±0.6
1-h IAH	Control	55±10	121±41	524±302	6±6	25±3	943±416	0.4±0.1
	Norepinephrine	53±9	118±42	633±383	12±5	27±3	552±359	0.4±0.7
	Sham	87±14*	120±28	449±88	4±6	24±3	1730±510*	0.9±0.5*
2-h IAH	Control	49±18*	134±39	522±322	8±6	24±2	869±612	0.3±0.4
	Norepinephrine	73±10	113±39	634±310	12±6	28±3	620±439	0.2±0.1
	Sham	87±15	127±24	448±108	3±5	25±4	1678±569*	1.0±0.6*

*p < 0.05 vs. the other groups (*t* test with Bonferroni correction after significant time x group interaction in two-way repeated measures of ANOVA).

## Conclusions

In this experimental model of IAH, the gut and the kidney displayed contrasting responses. While intestinal blood flow and villi microcirculation remained unchanged, renal perfusion and urine output were severely compromised. The increase in blood pressure with norepinephrine failed to improve these variables.

